# Multiple change point analysis of hepatitis B reports in Xinjiang, China from 2006 to 2021

**DOI:** 10.3389/fpubh.2023.1223176

**Published:** 2023-11-14

**Authors:** Liping Yang, Na Xie, Yanru Yao, Chunxia Wang, Ramziya RiFhat, Maozai Tian, Kai Wang

**Affiliations:** ^1^College of Public Health, Xinjiang Medical University, Ürümqi, China; ^2^College of Medical Engineering and Technology, Xinjiang Medical University, Ürümqi, China; ^3^Department of Immunization Programme, Xinjiang Center for Disease Control and Prevention, Ürümqi, China; ^4^College of Science, Shihezi University, Shihezi, China

**Keywords:** hepatitis B, change point analysis, binary segmentation, segmented regression, time series

## Abstract

**Objective:**

Hepatitis B (HB) is a major global challenge, but there has been a lack of epidemiological studies on HB incidence in Xinjiang from a change-point perspective. This study aims to bridge this gap by identifying significant change points and trends.

**Method:**

The datasets were obtained from the Xinjiang Information System for Disease Control and Prevention. Change points were identified using binary segmentation for full datasets and a segmented regression model for five age groups.

**Results:**

The results showed four change points for the quarterly HB time series, with the period between the first change point (March 2007) and the second change point (March 2010) having the highest mean number of HB reports. In the subsequent segments, there was a clear downward trend in reported cases. The segmented regression model showed different numbers of change points for each age group, with the 30–50, 51–80, and 15–29 age groups having higher growth rates.

**Conclusion:**

Change point analysis has valuable applications in epidemiology. These findings provide important information for future epidemiological studies and early warning systems for HB.

## 1. Introduction

Hepatitis B (HB) is a serious and prevalent disease caused by the hepatitis B virus (HBV) (1, 2). It is a leading cause of hepatic decompensation, cirrhosis, and hepatocellular carcinoma (3). According to the World Health Organization (WHO), in 2019, approximately 296 million people were living with chronic HB infections, and 1.5 million new infections occurred each year (4). HBV can be transmitted through blood and other bodily fluids, including saliva, tears, semen, and vaginal secretions. The most common modes of transmission include transmission from mother to child during delivery, unsafe injections, and sexual contact with an infected partner (4).

In China, HB has topped the list of category B infectious diseases in the annual report of nationally notifiable infectious diseases from the Chinese Center for Disease Control and Prevention. Xinjiang, located in the northwest of China, which is considered a high-prevalence area for HB (5–7). Therefore, conducting epidemiological studies in this region is critically important for the prevention and control of this disease.

The Change Point Analysis (CPA) method can be utilized to investigate whether one or more changes occur within a series of data points and if there are significant trends in time series data (8, 9). The CPA is widely applied in various scientific fields, including bioinformatics (10, 11), financial modeling (12), hydrology (13), climatology (14), and others. Notably, in the field of epidemiology, CPA can effectively quantify the burden caused by the infection of a particular disease and identify the outbreak of certain diseases (15), thus aiding in the implementation of suitable prevention and control measures to prevent further outbreaks in the field of epidemiology (16, 17), where the change points can elucidate the pattern and infection cycle of an epidemic (18).

To gain a better understanding of the epidemiology of HB, it is crucial to investigate the change points and trend changes in HB time series data. Therefore, this study aims to achieve three objectives: (i) to discuss the epidemiological characteristics of HB from the perspective of change points, (ii) to identify the change points and trend changes in the full HB time series data, and (iii) to identify the change points and trend changes in the time series data of five different age groups.

## 2. Materials and methods

### 2.1. Study area and data source

Xinjiang is located at 74°-96° east longitude and 34°-49° north latitude in the northwest of China and covers an area of approximately 1,664,900 km^2^. As of the end of 2022, the permanent resident population in Xinjiang amounted to 25.78 million. Our study utilized data on daily HB cases in Xinjiang (excluding the Xinjiang production and construction corps) from 1 January 2006 to 31 December 2021, which was obtained from the Xinjiang Information System for Disease Control and Prevention. This dataset contains information on the patient's age, place of residence, onset date of symptoms, time of symptom confirmation, and confirmation address of symptoms. Based on the daily onset time series, we generated monthly, quarterly, and annual onset time series. As the data used for this analysis did not involve any private patient information, no ethical approval or informed consent was deemed necessary.

### 2.2. The method of CPA

#### 2.2.1. The binary segmentation technique

The binary segmentation technique, a likelihood-based approach, was employed in order to detect the changes in the HB time series, where the count data of HB reports in Xinjiang is assumed to follow the Poisson distribution. Let *y*_1:*n*_ = (*y*_1_, …, *y*_*n*_) is a time series. A change point is said to occur in the datasets if there exists a time τ ∈ {1, …, *n*−1} lead to the differently statistical properties between {*y*_1_, …, *y*_τ_} and {*y*_τ+1_, …, *y*_*n*_}. Extending the idea to multiple change points, the number of *k* changes occur at τ_1:*k*_ ∈ {τ_1_, …, τ_*k*_}, and each change point position is an integer between 1 and *n* − 1 inclusive, where let τ_0_ = 0 and τ_*K*+1_ = *n* without loss of generality. The most common approach in identifying multiple change points is to minimize


(1) $$\begin{array}{ll} \sum_{i=1}^{k+1}\left[C\left(y_{\left(\tau_{i-1}+1:\tau_{i}\right)}\right)\right]+\beta f\left(k\right) & \;\left(1\right) \end{array}$$

where C is a cost function for a segment (e.g., negative log-likelihood) and β*f*(*k*) is a penalty to guard against overfitting (19). The *cpt.meanvar* function in the R package “changepoint” and the binary segmentation technique (19, 20) were utilized in order to search for multiple change points. The *cpt.meanvar* is a practical tool to detect both mean and variance changes. Binary segmentation is aimed at estimating an approximate minimum of Equation (1). Once the change points were identified, the corresponding segments could still be represented, such as the *i*th (*i* = 1, …, *k* + 1) segment, which could be found between the (*i* − 1)th and *i*th change points.

#### 2.2.2. Segmented regression model

The non-linear function estimates connected through two, three, or more straight lines at unknown points are referred to as change points, breakpoints, or join points. Let *y*_*t*_ be the cumulative number of reported HB cases at time *t* = 1, 2, …, *n*. A relationship between the mean response *E*(*y*_*t*_) and the explanatory variable *t* is explained by adding the linear term of the model. Then, the segmented model is described as follows:


(2) $$\begin{array}{ll} \text{log}E\left(y_{t}\right)=\alpha_{0}+\alpha_{1}t+\sum_{i=1}^{K}\gamma_{i}{\left(t-\delta_{i}\right)}_{+}. & \;\left(2\right) \end{array}$$

Here, we assume there are *K*+1 different regimes with slopes α_1_, α_2_ = α_1_+γ_*i*_, and $\alpha_{K}=\alpha_{1}+\overset{k}{\underset{1}{\sum }}\gamma_{i}$. Then, we calculate the percent growth rate by *r*_*k*_ = exp{α_*k*_}−1 for each segment *k* = 1, 2, …, *K*+1. In addition, we can also report the doubling time, *d*_*k*_ = log(2)/α_*k*_, also for each regime; this is a parameter to express the number of times requested to double the number of cases. All the model parameters, including the breakpoints, can be estimated by Poisson likelihoods or quasi-likelihoods (21). The Bayesian Information Criterion (BIC) can be used to choose a better model when several segmented models have been fitted with observed data. The analysis has been performed on R (version 4.3.0) using the “segmented package”.

## 3. Result

### 3.1. Data processing and analysis

The data for this study were recorded with EXCEL 2022, and the CPA process was performed by the R (version 4.3.0) software. The significant level is 0.05.

### 3.2. HB incidence reports

**Figure 1:**
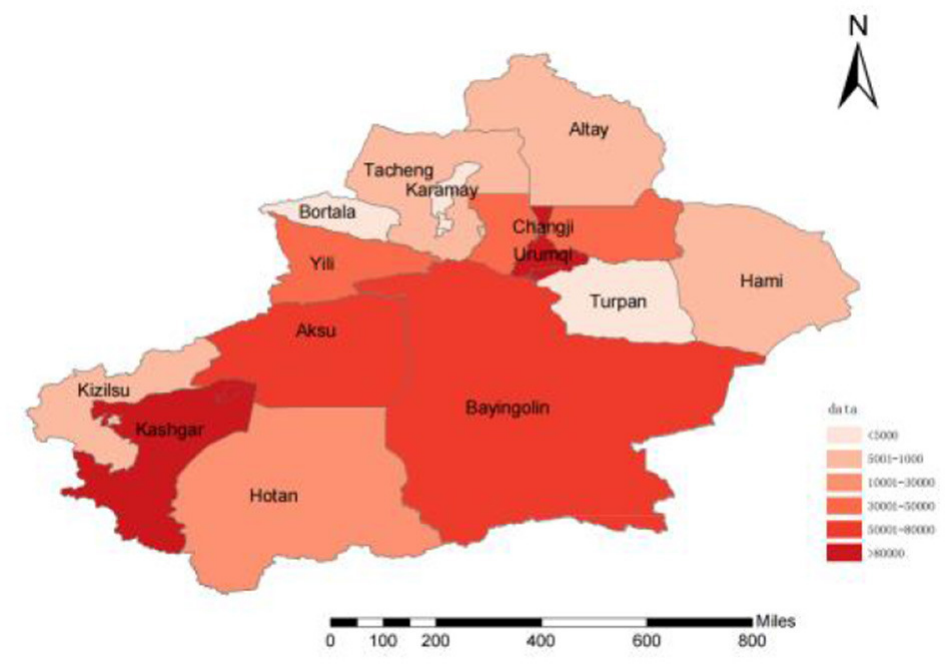
The study area and the number of HB reports of Xinjiang, China, from 2006 to 2021.

**Figure 2:**
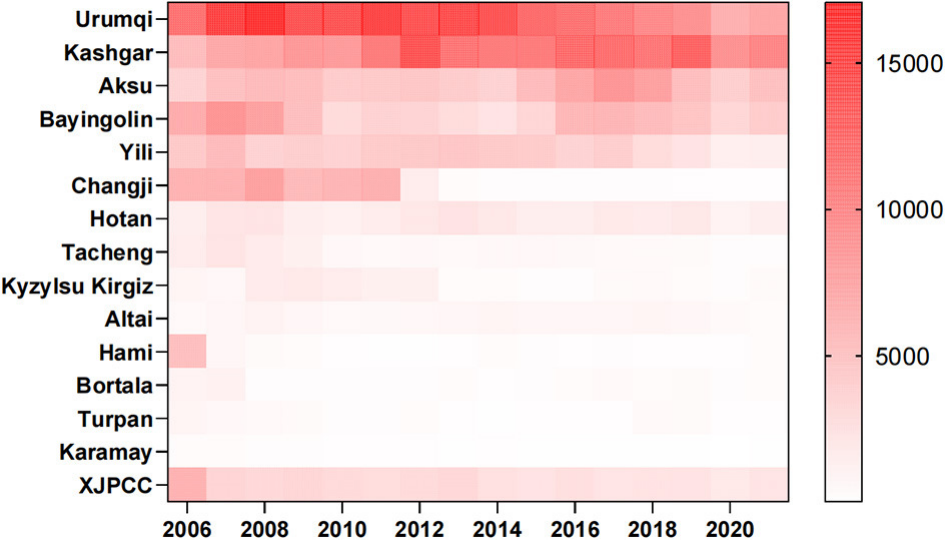
Annual cases of HB in each region of Xinjiang, China, from 2006 to 2021.

**Figure 3:**
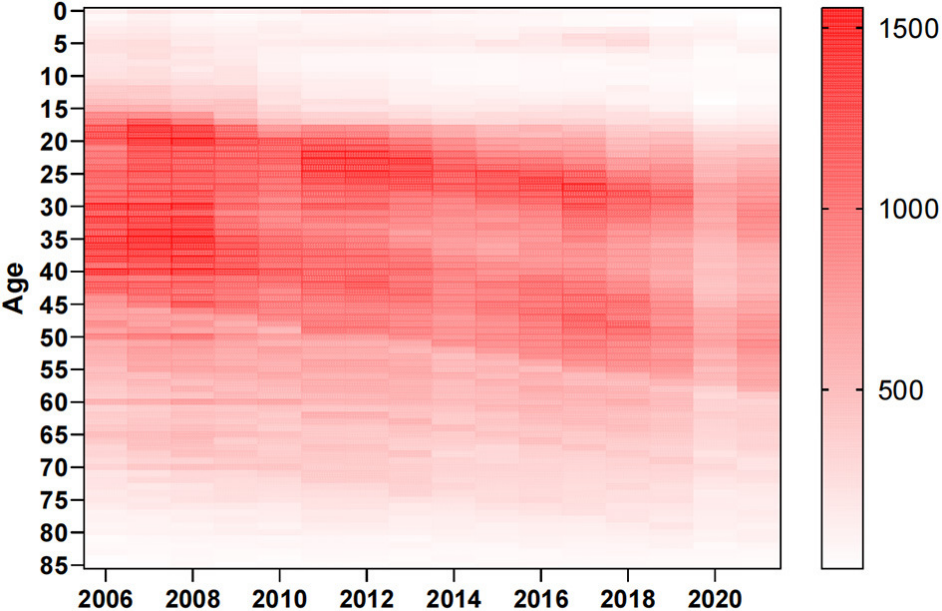
Annual cases of HB for all ages in Xinjiang, China, from 2006 to 2021.

**Figure 4:**
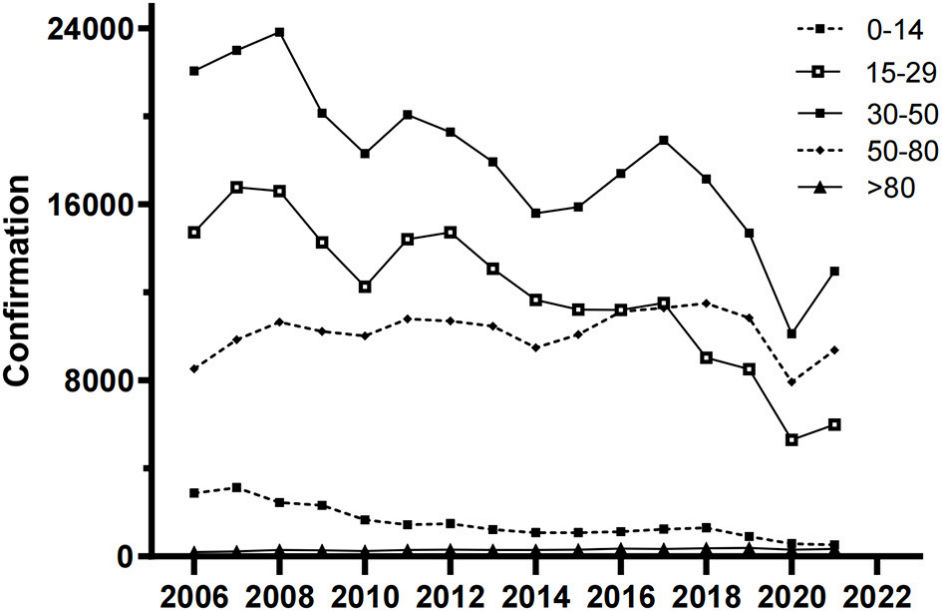
Annual cases of HB for five age groups in Xinjiang, China, from 2006 to 2021.

In total, 670,681 HB cases were reported from 1 January 2006 to 31 December 2021 in Xinjiang, China. Figures 1, 2 show the study area and annual cases of HB reported in each region of Xinjiang, China. These two figures indicated that there were more reports of HB in the southern and central regions of Xinjiang. In general, the cases of HB in each region showed a downward trend year by year, but the number of cases still undulated in some regions, like Urumqi, Kashgar, and Aksu. Figure 3 illustrates the incidence of HB across all age groups. It was evident from the figure that HB onset varies significantly by age. Therefore, the population was separated into five age groups (0–14, 15–29, 30–50, 50–80, and 80+), taking into account the age differences and HB prevention and control policies implemented in China. Figure 4 displays the cases of HB for five age groups in Xinjiang, China, from 2006 to 2021. Most HB cases typically occurred in groups 30–50, 15–29, and 50–80, the sum of cases (641,469) in these three groups exceeded 95.6% of the total cases. The cases in each group also showed a decreasing trend with several fluctuations.

### 3.3. Change points in the time series data of HB

**Figure 5:**
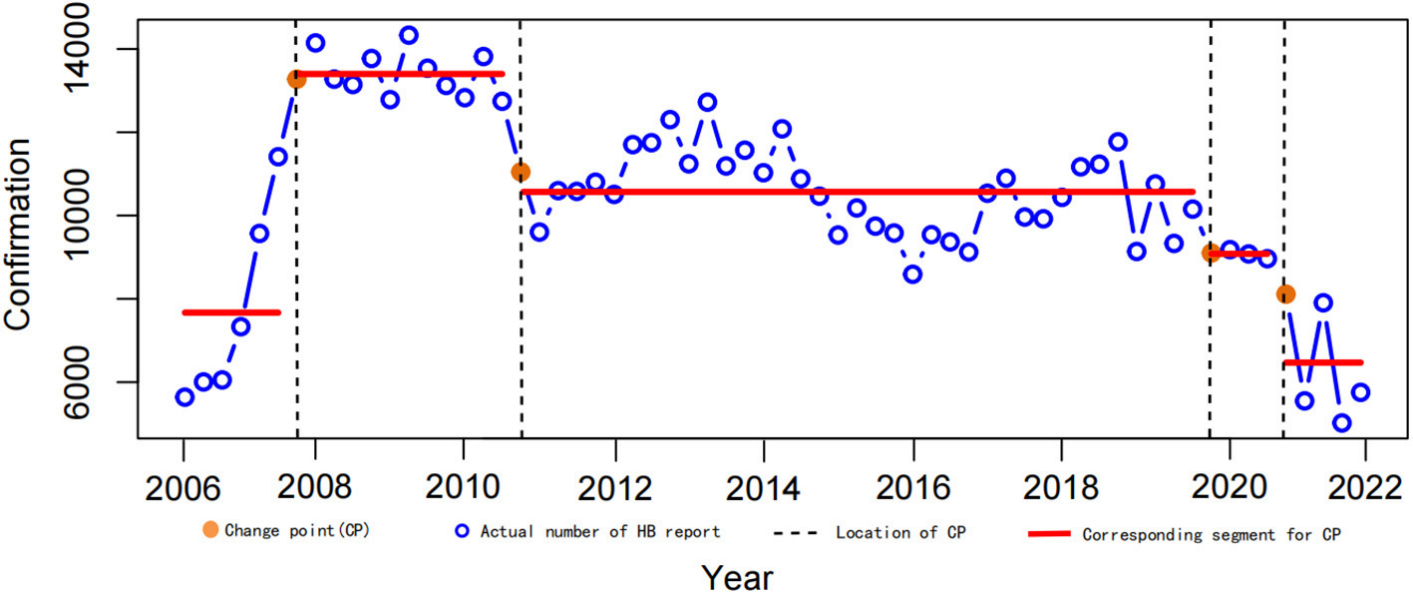
Change points in the time series of HB quarterly reports in Xinjiang, 2006–2021. Orange dots are change points, and red lines are corresponding segments.

In Xinjiang, the quarterly HB time series from 2006 to 2021 was analyzed, and four change points (orange dots) were detected using the binary segmentation technique. Five segments, corresponding to the change points, were identified (Figure 5). Each segment represents the mean values of the number of HB cases (red line) reported during the corresponding period. Compared with other segments, the second segment of the HB datasets had the highest mean number of cases (13,398 cases), and the last segment was the lowest (6,469 cases). The first change point occurred in March 2007, and there was a significant increase (74.7%) from the first to the second segment. Then, the cases of HB were stable at a high level between the first quarter of 2007 and the first quarter of 2010. After the second change point occurred (March 2010), the number of HB reports remained relatively stable for a long time until the second quarter of 2019. The third and fourth change points were detected in June 2019 and June 2020, respectively.

**Figure 6:**
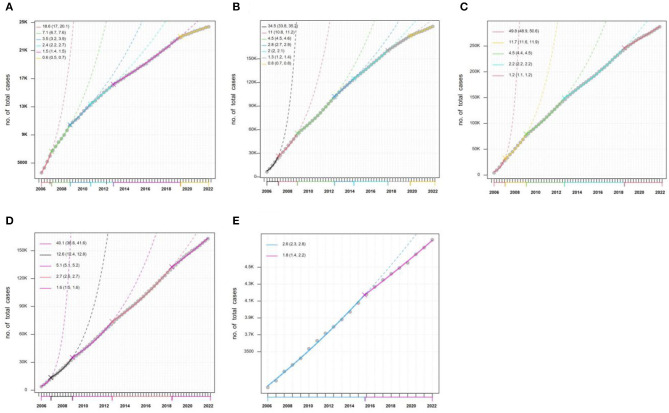
The results of CPA for the sequence of HB cumulative outcome counts in five (0–14, 15–29, 30–50, 50–80, and 80+) different age groups. **(A)** 0–14; **(B)** 15–29; **(C)** 30–50; **(D)** 50–80; **(E)** 80+.

Based on the segmented regression model, we split the sequence of HB cumulative counts into five (0–14, 15–29, 30–50, 50–80, and 80+) different age groups into several segments. Line segments were fitted for each interval to investigate the report cases between two change points. The objective of splitting is to analyze the change points, the growth rate, and doubling times within a specified period. The number of change points varied among age groups as the best model was selected based on the lowest BIC (bold values in Table 1), with the BIC values reported in Table 1. In Figure 6, the CPA results corresponding to Table 1 are presented, shown as connecting points linked by lines. These lines are considered segments where the trends are similar and are identified by different colors. The incidence rates with the 95% confidence interval (95% CI) are calculated for each segment and shown in Figure 6.

**Table 1:** The BIC value of CPA in each sequence of five age groups in Xinjiang.

**The number of change points**	**0–14**	**15–29**	**30–50**	**51–80**	**80+**
CP-1	2,397.51	42,062.56	15,475.91	6,100.43	**225.60**
CP-2	1,239.42	8,669.92	9,307.52	3,090.79	231.05
CP-3	945.93	4,398.93	2,853.85	1,403.56	237.07
CP-4	761.99	1,767.55	**1,756.90**	**1,146.31**	243.08
CP-5	**748.15**	1,530.39	1,780.09	1,176.53	249.13
CP-6	755.37	**1,514.42**	1,823.30	1,285.53	255.21

As seen in Figure 6A, five change points were detected in the age of 0–14 HB series. The incidence rate [18.6% (95% CI: 17%−20.1%)] was high in segment 1 (January 2006 to February 2007), and it gradually decreased to 7.1% (95% CI: 6.7%−7.6%), 3.5% (95% CI: 3.2%−3.9%), 2.4% (95% CI: 2.2%−2.7%), 1.5% (95% CI: 1.4%−1.5%), and 0.6% (95% CI: 0.5%−0.7%) in segment 2, segment 3, segment 4, segment 5, and segment 6, respectively. For other age groups, there was the same trend change in the datasets. But for the same segment in all age group, the more important information would be found. For example, there was the highest incidence rate [49.8% (95% CI: 48.9%−50.6%)] in the group of 30–50 (Figure 6C) in segment 1, and the lowest incidence rate in the last segment for all groups.

Moreover, doubling time (DT) is a parameter of interest that is often reported by epidemiologists and health professionals. Table 2 reports the DT with the 95% confidence interval (95% CI) for every segment. It is quite an easy-to-understand parameter that a shorter DT indicates a higher report of disease. It should be noted that the time series of quarterly HB data used here should take seasonal factors into account. It is found that the doubling times in the first segment are short for the five age groups. The results show a high incidence rate in this segment, especially for the groups of 30–50 and 51–80. However, the DT for each group tends to increase, which is undoubtedly a good signal in epidemiological terms.

**Table 2:** The doubling time and the 95% confidence interval (95% CI) of each segment for five age groups in Xinjiang.

**Doubling time *d*_*k*_**	**0–14**	**15–29**	**30–50**	**51–80**	**80+**
*d*_1_	4.07 (3.79,4.40)	2.34 (2.30,2.38)	1.72 (1.69, 1.74)	2.05 (1.99, 2.12)	27.34 (24.67, 30.67)
*d*_2_	10.07 (9.51,1.69)	6.65 (6.53, 6.77)	6.26 (6.19, 6.34)	5.84 (5.74, 5.94)	39.61 (32.58, 50.51)
*d*_3_	20.01 (18.23, 22.18)	15.71 (15.55, 15.88)	15.90 (15.76, 16.05)	13.81 (13.66, 13.95)	
*d*_4_	29.10 (26.35, 32.49)	25.24 (24.27, 26.29)	31.58 (31.37, 31.77)	26.33 (26.13, 26.55)	
*d*_5_	46.86 (45.58, 48.21)	34.72 (34.06, 35.40)	59.87 (58.59, 61.19)	44.42 (43.48, 45.41)	
*d*_6_	115.57 (93.71, 150.75)	53.43 (50.57, 56.63)			
*d*_7_		92.45 (85.74,100.32)			

In Table 3, we summarize the piecewise trends of different age groups by means of the average percent change, which is calculated as the average of the slopes weighted by the corresponding interval width (22). We report the average growth rate (*AGE* = *e*^*Est*.^−1) over the entire period. Here, the results show that the age groups of 30–50, 51–80, and 15–29 reflect the higher growth rate.

**Table 3:** The means of the average percent change (Est.) and the *AGE* for each sequence of the five age groups in Xinjiang.

	**0–14**	**15–29**	**30–50**	**51–80**	**80+**
Est.	0.0323	0.0523	0.0614	0.0593	0.0221
St. Err	0.0002	0.0001	0.0001	0.0002	0.0006
95% CI, lower	0.0318	0.0521	0.0611	0.0589	0.0208
95% CI, upper	0.0328	0.5259	0.0616	0.0596	0.0234
*AGE* (95% CI)	3.28% (3.23%, 3.33%)	5.37% (5.34%, 5.40%)	6.33% (6.30%, 6.35%)	6.10% (6.06%, 6.14%)	2.23% (2.10%, 2.37%)

## 4. Discussion

Despite the implementation of universal HBV vaccination programs in the 1990s, which has helped many countries significantly reduce the incidence of acute HB and the prevalence of chronic carriers of HB surface antigen (HBsAg), HB remains a major global public health concern. This is particularly true in developing countries and rural areas where HBV is widespread. With its vast territory and large population, the prevention and control of HB remain an important public health issue in Xinjiang, China. Detecting significant change points in the Xinjiang HB report time series provides valuable epidemiological information, particularly in the temporal dimension (23).

Our results provided fundamental information about HB, such as its geographic distribution and age composition. More HB cases were reported in the southern and central cities of Xinjiang. The high number of reported HB cases in southern Xinjiang might be related to low awareness and vaccination rates among the population, as well as inadequate or suboptimal medical management of HB cases. In Urumqi, the central city of Xinjiang, the high incidence might be attributed to various factors. As the capital city and economic and cultural center of Xinjiang, Urumqi attracted a large number of migrant workers, farmers, left-behind children, and other population groups with potentially high rates of HB infection. Additionally, it can also be attributed to the well-established HB testing and reporting systems in the region, which may ensure timely and accurate diagnosis and reporting. The declining trend of HB confirmation in several regions and age groups in Xinjiang (Figures 3, 4) might be related to the effectiveness of HB control measures and treatment programs in China (24).

The specific change points and trend changes were reported using the CPA approach (Figure 5). The results showed a significant increase in reported HB cases in Xinjiang from 2006 to 2007. This increase could be attributed to the gradual improvement of the disease surveillance system, which led to improved accuracy in data collection. In segment 2 (from March 2007 to March 2010) and segment 3 (from March 2010 to June 2019), the HB reports remained at a high level for a long time. This suggested that Xinjiang was indeed one of the areas with the highest incidence of HB. However, the numbers were generally declining from 2010 to 2021, which might be due to the improvement of health prevention awareness among the population and an increase in HB vaccination rates among the population. However, it was worth noting that blind optimism should not be attached to the low number of reported HB cases since 2020. Undoubtedly, the sudden global infectious disease, COVID-19, had a significant impact on the reported number of HB cases beginning in 2020 (25).

According to the results of the segmented regression model, the age groups of 30–50 and 51–80 experienced a higher growth rate, as shown by a smaller DT (Table 2) and higher values of *AGE* (Table 3). Unhealthy lifestyle choices, such as excessive workload, life pressures, frequent late nights, inappropriate work practices, and alcohol and tobacco consumption, may have contributed to this finding. The age group of 15–30 was also at a higher risk of HB reporting an increase due to various factors, including engaging in unsafe sexual behavior, having multiple sexual partners, reduced immune function, and a lack of awareness and knowledge regarding HB among young people (26). Moreover, in 1992, China began implementing a nationwide HB vaccination program. This policy mandated that infants receive their first dose of the HB vaccine within 24 h after birth (24, 27). As a result, children aged 0–14 years became beneficiaries of this program, which led to a significant decrease in the growth rate of HB. Therefore, it is critical to implement tailored strategies for the prevention and control of HB in different age groups.

For newborns, strict adherence to the administration of the HB vaccine is necessary. Young people should also adopt a healthy lifestyle to reduce the risk factors associated with the disease. Regular physical examinations for older adults can help identify the disease at its earliest stage. Moreover, health education on HB is pivotal for all age groups. The public should be well-informed about HB, including transmission and prevention measures. Health authorities should proactively raise awareness and understanding of the disease in communities, schools, and workplaces. Access to HB testing and treatment should be readily available. Health facilities should be adequately equipped with resources and trained healthcare professionals to diagnose and treat the disease. Affordable treatment options must be made available to ensure everyone has equal access to treatment. Additionally, continuous research on HB prevention and treatment is also crucial; this includes the development of new therapies and improved vaccines to better combat HB.

In summary, HB prevention and control involve a comprehensive approach targeting different age groups. It requires collaboration between health authorities, healthcare professionals, and the public. By implementing these recommendations, we can effectively reduce the burden of HB and ensure a healthy future for all. In the context of future HBV prevention and control, it is possible to utilize more precise change point detection methods to study the epidemiological characteristics of HBV itself. Furthermore, the analysis can be conducted to evaluate the effectiveness of disease prevention and control measures.

## 5. Conclusion

Investigating change points and trend changes can facilitate informed decisions regarding the prevention of further disease outbreaks, as they can elucidate the pattern and infection cycle of an epidemic. Despite reports of HB decreasing during the CPA process, it persists as a significant public health problem in Xinjiang. As China undergoes rapid development, the social circles of young people are expanding, and unhealthy lifestyle choices such as smoking, irregular rest schedules, and drinking are becoming more prevalent. These behaviors may contribute to the burden of HB and increase its incidence rate in the future. We therefore urge local public health departments to prioritize the prevention and control of HB. Society as a whole should promote healthier lifestyles, and supervision of HB vaccination for newborns in rural and remote areas should remain a focus. Furthermore, the study highlights the differing growth rates of HB among age groups, further emphasizing the significance of adopting age-specific prevention and control measures to mitigate its transmission and spread.

## Data availability statement

The datasets presented in this article are not readily available because the data that support the findings of this study are available from the corresponding author upon reasonable request. Requests to access the datasets should be directed to KW, wangkaimath@sina.com.

## Author contributions

LY: writing—original draft, data analysis, and obtained funding. NX and KW: data curation, data analysis, reviewing, and editing. YY, CW, and RR: data analysis and revised manuscript. MT: conception, reviewing, and editing. All authors contributed to the article and approved the submitted version.

## References

[1] Razavi-Shearer D Gamkrelidze I Nguyen MH Chen D-S Damme PV Abbas Z. Global prevalence, treatment, and prevention of hepatitis B virus infection in 2016: a modelling study. Lancet Gastroenterol Hepatol. (2018) 3:383–403. 10.1016/S2468-1253(18)30056-6 29599078

[2] Maynard JE. Hepatitis B: Global importance and need for control. Vaccine. (1990) 8:S18–20. 10.1016/0264-410X(90)90209-5 2139281

[3] Nelson N P Easterbrook P J Mcmahon B J. Epidemiology of hepatitis b virus infection and impact of vaccination on disease. Clin Liver Dis. (2016) 20:607–28. 10.1016/j.cld.2016.06.006 27742003 PMC5582972

[4] WHO. Hepatitis B Fact Sheets. Available online at: https://www.who.int/news-room/fact-sheets/detail/hepatitis-b (accessed April 4, 2023).

[5] Jiang W Mao XH Liu ZQ. Analysis of the incidence trend of hepatitis B in Chinese residents from 2004 to 2017. Chin J Public Health. (2022) 38:257–61. 10.11847/zgggws1132715

[6] Ji W Xie N He D Wang W Li H Wang K. Age-period-cohort analysis on the time trend of hepatitis B incidence in four prefectures of southern Xinjiang, China from 2005 to 2017. Int J Environ Res Public Health. (2019) 16:3886. 10.3390/ijerph16203886 31615013 PMC6843167

[7] Wang Y Xie N Li F Wang Z Ding S Hu X. Spatial age-period-cohort analysis of hepatitis B risk in Xinjiang from 2006 to 2019. Front Public Health. (2023) 11:1171516. 10.3389/fpubh.2023.1171516 37325304 PMC10264624

[8] Gargoum SA Gargoum AS. Limiting mobility during covid-19 when and to what level? An international comparative study using change point analysis. J Transp Health. (2021) 20:101019. 10.1016/j.jth.2021.101019 33777694 PMC7984960

[9] Kass-Hout TA Xu Z McMurray P Park S Buckeridge DL Brownstein JS. Application of change point analysis to daily influenza-like illness emergency department visits. J Am Med Inform Assoc. (2012) 6:1075–81. 10.1136/amiajnl-2011-000793 22759619 PMC3534458

[10] Shen JJ Zhang NR. Change-point model on nonhomogeneous poisson processes with application in copy number profiling by next-generation DNA sequencing. Ann Appl Statist. (2012) 6:476–96. 10.1214/11-AOAS517

[11] Muggeo V Adelfio G. Efficient change point detection for genomic sequences of continuous measurements. Bioinformatics. (2011) 2:27. 10.1093/bioinformatics/btq647 21088029

[12] Teyssière G Kirman AP. Long Memory in Economics. Berlin: Springer Berlin Heidelberg. (2007).

[13] Cao X Wang J Liao J Gao Z Jiang D Sun J. Bacterioplankton community responses to key environmental variables in plateau freshwater lake ecosystems: a structural equation modeling and change point analysis. Sci Total Environ. (2017) 580:457–67. 10.1016/j.scitotenv.2016.11.143 28040220

[14] Shi K Touge Y. Identifying the shift in global wildfire weather conditions over the past four decades: An analysis based on change-points and long-term trends. Geoscience Letters. (2023) 10:1–16. 10.1186/s40562-022-00255-636619610

[15] Texier G T Farouh M Pellegrin L Jackson ML Meynard J Deparis X. Outbreak definition by change point analysis: a tool for public health decision? BMC Med Inform Decis Mak. (2016) 16:33. 10.1186/s12911-016-0271-x 26968948 PMC4788889

[16] Chen X Wang H Lyu W Xu R. The mann-kendall-sneyers test to identify the change points of covid-19 time series in the united states. BMC Med Res Methodol. (2022) 22:1–9. 10.1186/s12874-022-01714-6 36042407 PMC9424808

[17] Punyapornwithaya V. Lumpy skin disease outbreaks in Africa, Europe, and Asia (2005–2022): multiple change point analysis and time series forecast. Viruses. (2022) 14:2203. 10.3390/v14102203 36298758 PMC9611638

[18] Pk. ST, Lahiri B, Alvarado R. Multiple change point estimation of trends in covid-19 infections and deaths in india as compared with who regions. Spatial Statist. (2021) 49:100538. 10.1016/j.spasta.2021.100538 PMC8413104 34493970

[19] Killick R Eckley IA. Changepoint: an r package for changepoint analysis. J Statist Softw. (2015) 58, 3. 10.18637/jss.v058.i03

[20] Constantinescu P. A method of cluster analysis. Br J Mathemat Statist Psychol. (2011) 20. 10.1111/j.2044-8317.1967.tb00381.x

[21] Muggeo V Sottile G Porcu M. Modelling COVID-19 Outbreak: Segmented Regression to Assess Lockdown Effectiveness. (2020).

[22] Muggeo V. Comment on 'estimating average annual per cent change in trend analysis' [mr2751730]. Stat Med. (2010) 29:1958–60. 10.1002/sim.3850 20680988

[23] Shaukat M. H, Alotaibi N, Hussain I, Shrahili M. The Analysis of the Incidence Rate of the COVID-19 Pandemic Based on Segmented Regression for Kuwait and Saudi Arabia. London: Hindawi Limited. (2021). 10.1155/2021/2644506

[24] Liu J Liang W Jiang W Liu M. Countdown to 2030: Eliminating hepatitis B disease, China. Bull World Health Organ. (2019) 97:230–8. 10.2471/BLT.18.219469 30992636 PMC6453311

[25] Zhang YQ Zhang HF. Study on the public medical treatment behavior under the COVID-19 epidemic. Med Soc. (2021) 34:7–11. 10.13723/j.yxysh.2021.07.002

[26] Franco E Bagnato B Marino M Meleleo C Serino L Zaratti L. Hepatitis B: Epidemiology and prevention in developing countries. World J Hepatol. (2012) 4:74–80. 10.4254/wjh.v4.i3.74 22489259 PMC3321493

[27] Meng J Xu H Sui D Jiang J Li J Gao Y. retrospective serological survey of hepatitis B virus infection in Northeast China. BMC Infect Dis. (2019) 19:440. 10.1186/s12879-019-4091-3 31109300 PMC6528233

